# Comparative analysis of plant transient expression vectors for targeted N-glycosylation

**DOI:** 10.3389/fbioe.2022.1073455

**Published:** 2022-12-21

**Authors:** Lukas Eidenberger, Florian Eminger, Alexandra Castilho, Herta Steinkellner

**Affiliations:** Department of Applied Genetics and Cell Biology, Institute of Plant Biotechnology and Cell Biology, University of Natural Resources and Life Sciences, Vienna, Austria

**Keywords:** Nicotiana benthamiana, transient expression, N-glycosylation, plant biotechnology, glycoengineering, IgG1

## Abstract

While plant-based transient expression systems have demonstrated their potency to rapidly express economically feasible quantities of complex human proteins, less is known about their compatibility with posttranslational modification control. Here we investigated three commonly used transient expression vectors, pEAQ, magnICON and pTra for their capability to express a multi-component protein with controlled and modified N-glycosylation. Cetuximab (Cx), a therapeutic IgG1 monoclonal antibody, which carries next to the conserved Fc an additional N-glycosylation site (GS) in the Fab-domain, was used as model. While pEAQ and pTra produce fully assembled Cx at similar levels in *N. benthamiana*, the yield of magnICON-Cx was twice as high. When expressed in wild type plants, both Cx-GSs exhibited typical plant N-glycans decorated with plant-specific xylose and fucose. Likewise, Cx generated in the glycoengineered ΔXTFT line carried mainly complex N-glycans lacking plant specific residues. Exposure to different engineering settings (encompassing stable lines and transient approaches) towards human galactosylation and sialylation resulted in Cx carrying targeted N-glycans at similar quantities using all three expression vectors. Collectively, our results exhibit the universal application of plant-based glycoengineering, thereby increasing the attractivity of the ambitious expression platform.

## 1 Introduction

Monoclonal antibodies (mAbs) are one of the most successful bio-pharmaceutical products. It has been estimated that the increasing demand may soon exceed the current production capacities (Ecker and Seymour, 2020; Kaplon et al., 2022), pointing to the need of production systems not only to increase expression but also to enhance efficacy. One platform with high potential is plant-based transient expression, allowing protein expression within days post DNA construct delivery, with product yields ranging in g/kg leaf material. Generally, four plant-based transient expression vectors are frequently used: pTra and pEAQ, where expression is mainly driven by strong promoters and viral translational enhancers (Sainsbury et al., 2009; Saxena et al., 2016; van Dolleweerd et al., 2018; Pinneh et al., 2022), magnICON, which is based on deconstructed virus genomes (Gleba et al., 2005; Giritch et al., 2006) and the Gemini virus-based BeYNV vector (Diamos et al., 2020).

Prototypes of industrial processes that provide economic yield, rapid scale up and fast manufacturing cycles have been designed and GMP-certified production facilities are in place [Reviewed (Schillberg and Finnern, 2021; Swope et al., 2022)]. The ability of plants to produce functionally active, recombinant mAbs has been demonstrated in manifold studies using the transient approach [recently reviewed (Chen, 2022)]. While mAb expression levels are reported in detail for the individual systems, comparative analyses are missing. Also, relatively little attention is paid to the characterization and engineering of posttranslational modifications (PTMs), like N-glycosylation. Control of N-glycosylation is not only important for product homogeneity but also for efficacy [reviewed (Nimmerjahn and Werner, 2021)]. Individual studies, mainly using magnICON vectors, indicate the capacity to generate mAbs with designed PTMs, thereby improving their activities [e.g., (Zeitlin et al., 2011; Loos et al., 2015; Kallolimath et al., 2020)]. However, a comparative evaluation of transient vectors would be beneficial to further judge their potencies and help researchers and companies to select the correct tools for their target proteins.

Here we aimed to evaluate three commonly used transient expression vectors (magnICON, pTra/MIDAS, and pEAQ) for their ability to express a monoclonal antibody and compatibility to have its N-glycosylation profile engineered. Note, all three vectors share features but also differ significantly in other aspects, such as i) ability of multi-gene expression; ii) p19 silencing suppressor co-expression; iii) amount of viral sequences encoded (Supplemetry Figure S1). Cetuximab (Cx, brand name Erbitux), was used as model mAb, since it carries, besides the conserved fragment crystallizable (Fc) N-glycosite (GS), another one at the heavy chain fragment antigen-binding (Fab) domain. It is well known that the two sites are differentially glycosylated (Bondt et al., 2014; Castilho et al., 2015), thus this mAb provides a particularly useful model. Identical Cx heavy (HC) and light chain (LC) open reading frames (ORF) were cloned into the three vector backbones and transiently expressed side by side in WT and different glycoengineering settings. Recombinant expression was comparatively monitored for mAb yields and biochemical features, with a special focus on N-glycosylation.

## 2 Materials and methods

### 2.1 Vector construction

Cx HC and LC ORFs (HC 1350 bp, LC 645 bp), codon optimized for *N. benthamiana*, were fused to barley α-amylase signal sequence (GenBank: X15226.1) (Supplementry Figure S3) and subcloned into the magnICON (vectors pICH26211 and pICH31150), pEAQ (vector pEAQ-HT) and pTra (vector pMIDAS) plasmids (Supplemetry Figure S1; Supplementry Table S2). All vectors were transformed into *Agrobacterium tumefaciens* (magnICON, pEAQ: strain GV3101 pMP90; pTra: GV3101 pMP90RK).

### 2.2 Agroinfiltration

*A. tumefaciens* strains carrying the respective constructs were propagated overnight in YEB-medium (1 g/L yeast extract, 5 g/L meat extract, 5 g/L peptone, 5 g/L sucrose, 0.24 g/L MgSO_4_; pH 7). Cells were harvested by centrifugation (2,348 g, RT, 5 min) and resuspended in the same volume of infiltration buffer (10 mM MES pH 5.7, 10 mM MgSO_4_). The cell density of the suspension was measured by extinction at 600 nm (OD_600_) of an adequate dilution. Suspensions were mixed and diluted with infiltration buffer to a final OD_600_ of either 0.1 for strains carrying mAb-ORFs or 0.05 for strains carrying ORFs of glycosylation enzymes (either *GalT* (modified human β-1,4-galactosyltransferase, for construct details see (Strasser et al., 2009)) or *FUT11* (modified *Zea mays* α-1,3-fucosyltransferase, for details see (Castilho et al., 2015)). *N. benthamiana* plants were grown in a plant chamber at 24°C, 60% humidity with a 16 h light/8 h dark photoperiod. Leaves of 4 to 5 weeks old plants were used for infiltration by syringe. Up to three fully expanded leaves of medium age were infiltrated per plant.

### 2.3 SDS-PAGE, immunoblotting, and purification

Infiltrated leaves were flash-frozen in liquid nitrogen and the cells disrupted by milling (Retsch MM 400). Total soluble proteins were extracted (0.1 M Tris, 0.5 M NaCl, 1 mM EDTA, 0.04 M ascorbic acid; pH 7) and SDS-PAGE analysis was performed in 12% gels under reducing or non-reducing conditions. Gels were stained with Coomassie Brilliant Blue R 250 or used for immunoblotting with anti-human IgG (1:5,000 Promega anti-hIgG-HRPO, W4031). Recombinant Cx was purified by affinity chromatography using protein A, eluted (0.1 M Glycine/HCl; pH 2.5) and neutralized (1 M Tris; pH 9). Protein concentration was measured by UV-Vis Spectrophotometer (NanoDrop™).

### 2.4 Glycan analysis

The N-glycosylation profiles of purified Cx-Fc and -Fab sites were determined by mass spectrometry (MS) as described previously (Kallolimath et al., 2020). In brief, the HC was extracted from an SDS-PA-gel, trypsin digested and analyzed with a LC-ESI-MS system (maXis 4G ETD, Bruker). Glycopeptides were identified as sets of peaks originating from the masses of the peptides and the attached N-glycan moieties, varying in the number of N-acetylhexosamine (GlcNAc), hexose (mannose, galactose), deoxyhexose (fucose), pentose (xylose) as well as Neu5Ac residues. Heights of peaks roughly reflect the molar ratios of the glycoforms relative to each other. Glycan nomenclature according to Consortium for Functional Glycomics (https://www.functionalglycomics.org) was used.

## 3 Results

**FIGURE 1:**
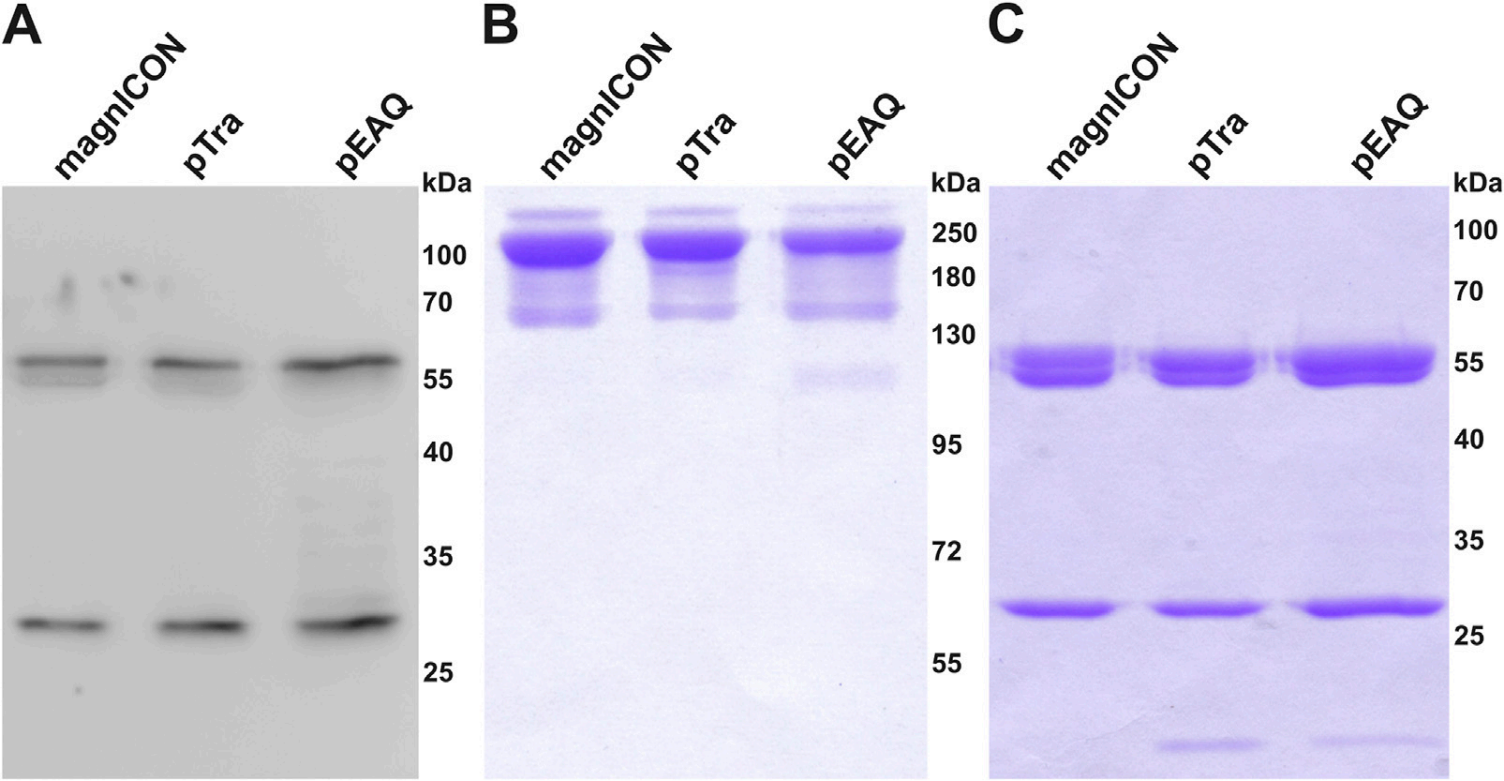
Expression and purification of Cetuximab (Cx). **(A)** Western blot analysis of total soluble protein extracted from WT plants infiltrated with magnICON-, pTra- and pEAQ-Cx (extracts were concentration-adjusted according to Cx yield to achieve comparable signal intensities); **(B)** SDS-PAGE of purified magnICON-, pTra- and pEAQ-Cx under non-reducing, and **(C)** reducing conditions. 3.5 μg IgG1 was loaded in each lane in both **(B)** and **(C)**. HC double bands represent glycosylated and non-glycosylated portions of the mAbs (Castilho et al., 2018). Low-molecular bands in **(C)** are presumably minor amounts of degradation products. Bands in **(B)** have overestimated weights due to a known issue with IgG unfolding, see (Kirley et al., 2018; Kirley and Norman, 2018) for details.

**FIGURE 2:**
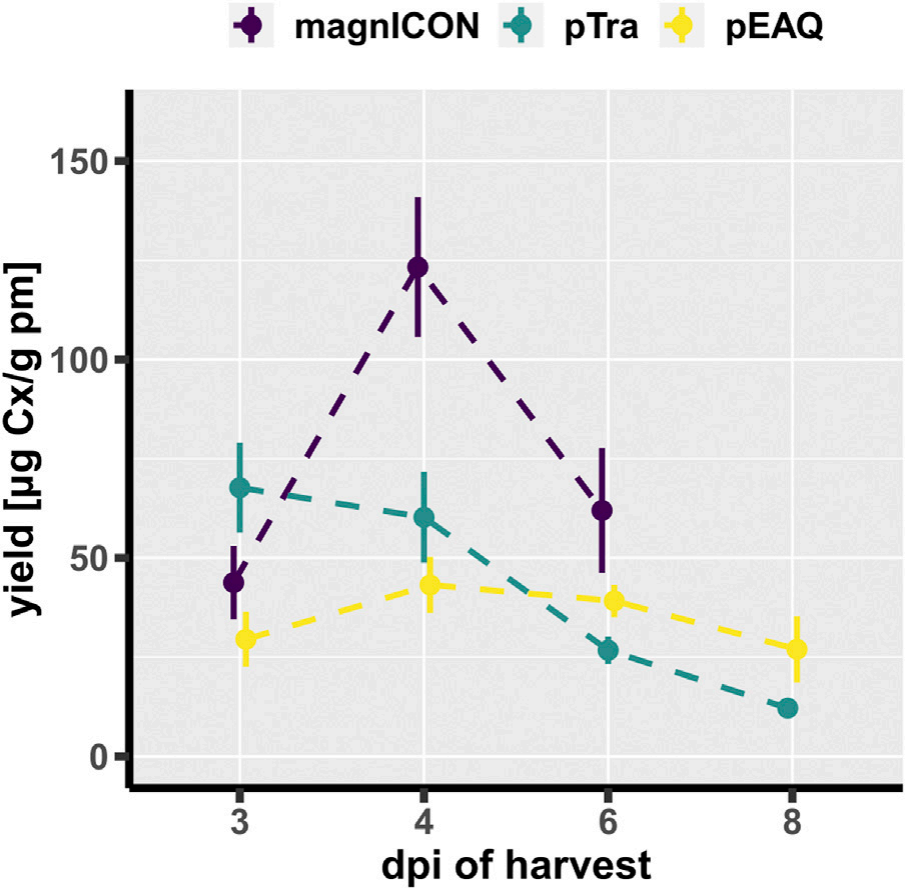
Yield of Cx at different days post infiltration (dpi), performed in triplicates. Error bars represent standard error of the mean. Yield in µg purified Cx per g plant material (pm).

**FIGURE 3:**
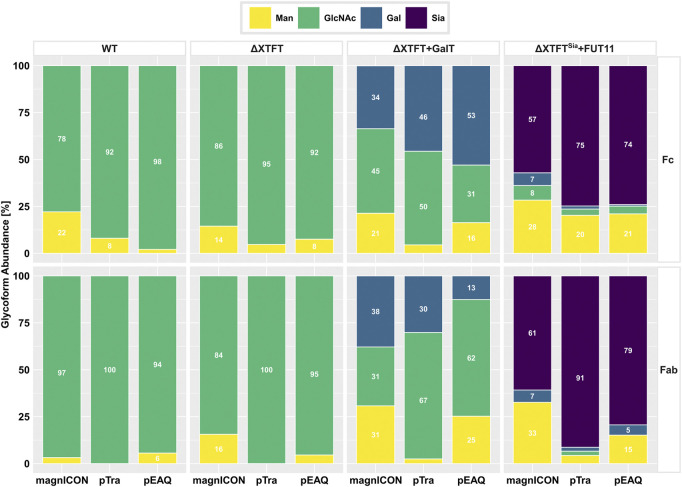
MS-based analyses of N-glycosylation of Cx produced by magnICON, pTra and pEAQ using *N. benthamiana* WT (WT), RNAi line ΔXTFT (ΔXTFT), ΔXTFT co-infiltrated with human *GalT* (ΔXTFT + GalT) and ΔXTFT^Sia^ (transgenic ΔXTFT, stably transformed with human sialylation pathway) and *FUT11* co-infiltration (ΔXTFT^Sia^ + FUT11). Glycans are grouped as follows: yellow: mannosidic structures; green: GlcNAc terminating N-glycans; blue: galactosylated N-glycans; purple: sialylated N-glycans. Bars represent the relative abundance (%) of glycoforms present at the Fc and the Fab domain, respectively (for detailed information, see also Supplmentary Figure S2). Abundances ≥5% are displayed as rounded whole numbers.

Cx, a clinically approved human IgG1 mAb, applied in a variety of epidermal growth factor receptor-overexpressing cancer types, served as model in this study. This mAb was chosen because it contains, next to the conserved Fc-site, an additional N-glycosite at the HC Fab domain, which is present in about 20% of serum IgGs (Anthony et al., 2012). Both sites are differentially N-glycosylated, thus this molecule provides a good example to trace N-glycosylation (Castilho et al., 2015). Identical HC- and LC-ORFs of Cx were cloned into the three transient expression vectors magnICON (Gleba et al., 2005; Giritch et al., 2006), MIDAS (van Dolleweerd et al., 2018; Pinneh et al., 2022) and pEAQ (Sainsbury et al., 2009; Saxena et al., 2016). The MIDAS vector is based on a pTra backbone and will henceforth be referred to as pTra. While magnICON and pEAQ carry the two Cx-ORFs on separate plasmids, pTra contains both on one plasmid, as this vector is designed to house multiple expression cassettes (Supplemetry Figure S1). Wild type (WT) *N. benthamiana* leaves were infiltrated with the respective agrobacteria (OD_600_ 0.1), either with one (pTra) or two (magnICON, pEAQ) strains. Infiltrated leaf material was harvested 4 days post infiltration (dpi) and Cx expression monitored by western blotting using labelled anti-human IgG for detection. A similar banding pattern was observed for all three vectors; next to bands with molecular masses corresponding to HC and LC (50 kDa and 25 kDa, respectively), no further or minimal others were detected (Figure 1A). Cx was purified by immuno-affinity means and monitored by SDS-PAGE. Under reducing conditions, Cx originating from all three vectors exhibited two major bands corresponding to the molecular mass of the IgG1 HC and LC, respectively (Figure 1C). No obvious degradation products were detected. Non-reducing SDS-PAGE showed a predominant signal at molecular mass >160 kDa (Figure 1B), indicating full assembly. To establish a temporal trend of mAb yield, infiltrated leaves were harvested at four timepoints post infiltration (3, 4, 6 and 8 dpi) and purified Cx was quantified by UV-Vis extinction. pTra- and pEAQ-Cx exhibited similar yields, with a peak at 3 dpi and 4 dpi, respectively (Figure 2). magnICON-Cx yield peaked at 4 dpi as well, but showed a substantially higher yield both at 4 dpi and 6 dpi compared to the other two vectors. Note, using OD_600_ 0.1 for infiltration of magnICON constructs induced leaf necrosis already 4-5 dpi, which impeded harvesting beyond 6 dpi, in contrast to pEAQ and pTra, where necrosis was not observed. The N-glycosylation status of Cx produced with the three vectors was determined by mass spectrometric methods. Both GSs exhibited fully processed complex N-glycans, namely GnGnXF structures up to 95% (Figure 3), typical for IgG1s produced in WT plants (Strasser et al., 2008). Collectively, the observed results point to the ability of all three vectors to produce Cx with no obvious differences with regards to biochemical features.

Next, we determined the susceptibility of Cx, originating from the three expression vectors, to glycoengineering. We have previously shown that proteins produced with magnICON vectors are amendable to various glycoengineering settings, either using stable transgenic lines or transient engineering by the co-expression of diverse glycosylation enzymes (Strasser et al., 2009; Kallolimath et al., 2016; Montero-Morales and Steinkellner, 2018). Here, Cx was produced with the three vectors, each in three different settings: using i) *N. benthamiana* glycosylation mutant ΔXTFT, an RNAi line with downregulated xylosyl- and core α-1,3-fucosyltransferases (Strasser et al., 2008), ii) transient co-expression of a modified human β-1,4-galactosyltransferase (*GalT*) in ΔXTFT (Strasser et al., 2009) and iii) ΔXTFT^Sia^, a stable transgenic line carrying genes for the expression of the human sialylation pathway (Kallolimath et al., 2016). Additionally, plant α-1,3-fucosyltransferase (*FUT11*) was transiently co-expressed in iii), as Fc-sialylation is promoted by core fucose (Castilho et al., 2015). Infiltrated leaves were harvested 4 dpi and purified Cx subjected to MS analyses. Using ΔXTFT, all three expression vectors produced Cx carrying a single dominant glycoform, namely GnGn structures, at both GSs, as typical for ΔXTFT produced IgG1s (Strasser et al., 2008) (Figure 3). While at the Fc-GS only fucose-free structures were present, up to 15% fucosylated N-glycans were detected at the Fab-GS, an observation already made earlier using ΔXTFT (Castilho et al., 2015).

Transient co-expression of *GalT* in ΔXTFT resulted in the generation of various degrees of galactosylated N-glycans. Depending on the expression vector, Fc-GSs exhibited 34–53% galactosylated structures, while this glycan formation was only detected 13–38% at the Fab domain (Figure 3). It should be noted that galactosylation is difficult to control, mainly due to the presence of highly active galactosidases *in planta* (Kriechbaum et al., 2020). The Fab-GS seems to be even more exposed to such activities compared to the “buried” Fc-GS, especially exemplified in pEAQ-Cx, with total galactosylation of 53% at the Fc-GS, but only 13% at the Fab-GS (Figure 3).

Cx expressed in ΔXTFT^Sia^ plants exhibited the following N-glycosylation profiles: sialylation efficiencies were between 57–75% on the Fc-GS and between 61–91% on the Fab-GS. Due to the above described fucosyltransferase co-expression, the majority of complex N-glycans (>90%) were fucosylated (Supplementary Figure S2). It was also observed that by increasing the number of foreign glycosylation enzymes, the complexity of the glycoprofile increased, highlighted by the amount of low abundance glycoforms (defined as <3.5%). These forms were preferentially detected at the Fc-GS and accounted for up to 27% glycoform abundance (Supplementary Figure S2). Collectively, all three expression vectors were found to be principally compatible with glycan engineering processes. However, it should be noted that magnICON-Cx showed some deviations in N-glycosylation compared to pTra and pEAQ. These included an 10–15% increase of mannosidic forms and incompletely processed galactosylated and sialylated structures (Supplementary Figure S2).

An interesting observation was made regarding GS occupancy. While the Fab was virtually 100% N-glycosylated in all settings, Fc-GS was only partially occupied, namely about 50% using the magnICON vector and approx. 70% using pTra and pEAQ (Supplementary Table S3). It seems that the Fab-GS is more accessible for the oligosaccharyltransferase complex that initiates N-glycosylation than its Fc counterpart, in accordance with results described above.

## 4 Discussion

The recent SARS-CoV2 outbreak advanced biotechnology-related research and industry in an unprecedented manner and made it clearer than ever before that biopharmaceutically related products are key components to efficiently approach current medical-associated challenges. Plant-based transient expression significantly contributed to the rapid supply of high-quality products for various applications, including therapeutics, vaccines, and diagnostic items (Shanmugaraj et al., 2020; Castilho et al., 2021; Jugler et al., 2021; Shin et al., 2021; Sun et al., 2021; Hager et al., 2022). The bi-N-glycosylated multi component protein Cetuximab provides a good model to assess the three potent transient expression vectors. By applying agrobacteria concentrations of OD_600_ 0.1, the yield peak of Cx was obtained in all three cases 3-4 dpi. However, expression level was about double using magnICON, demonstrating its outstanding power. The comparable Ab yields produced by the other two vectors correlated with their architectural similarities in terms of viral enhancer utilization to drive expression, in contrast to magnICON’s reconstructed viral genomes, but they also differ in other aspects. Therefore, claims on the effect of specific vector elements cannot be made based on the presented data. Importantly, we did not observe obvious differences in biochemical features as determined by SDS-PAGE under reducing and non-reducing conditions between Cx produced through the three vectors. Moreover, Cx expressed with all three expression vectors are similarly susceptible to glycoengineering. Interestingly, the downregulation of fucose and xylose using ∆XTFT remains stable. This is especially remarkable when using pEAQ, since this expression vector carries the p19 silencing suppressor gene, potentially weakening/releasing RNAi-based downregulation.

Compared to the other two, magnICON-Cx showed slightly enhanced mannosidic and incompletely processed galactosylated/sialylated structures (10–15%). Incomplete processing might be a consequence of high expression levels interfering with the secretory pathway. This inverse relationship of expression level and product quality and could be approached by optimization strategies, like determination of more appropriate agrobacteria concentrations. Note, compared to other research groups working with the same expression vector [e.g., (Zeitlin et al., 2011)], we used a relatively high OD_600_. Another unusual observation is the relatively low Fc-GS glycan occupancy, particularly pronounced at magnICON-Cx (approx. 50%). Although Fc-underglycosylation is frequently observed in plant produced mAbs (Castilho et al., 2018), it might be promoted by stressing the secretory pathway with a comparatively high expression. This phenomenon could be a consequence of insufficient activity of the plant inherent oligosaccharyltransferase complex that is responsible for the *en bloc* transfer of the oligosaccharide (Glc_3_Man_9_GlcNAc_2_) to nascent polypeptides. This shortcoming might be overcome by the overexpression of heterologous oligosaccharyltransferase domains, previously shown to increase GS occupancies (Castilho et al., 2018). Interestingly, GS occupancy seems to be highly site-specific, as Fab domains are virtually fully N-glycosylated in all cases.

Collectively, our results point to the enormous potential and the universal use of plant-based glycoengineering and may encourage more scientists to generate complex glycoproteins with designed PTMs. This improves product quality and efficacy, thereby increasing the attractivity of the so far highly underestimated plant-based expression systems.

## Data Availability

The raw data supporting the conclusions of this article will be made available by the authors, without undue reservation.
