# Supplementary material for: Comparative analysis of plant transient expression vectors for targeted N-glycosylation
Source: Front Bioeng Biotechnol. 2022 Dec 21;10:1073455. doi: 10.3389/fbioe.2022.1073455 (PMC9812561; doi:10.3389/fbioe.2022.1073455)
Supplement: Supplementary file 1 [file DataSheet1.PDF]

Supplementary Data

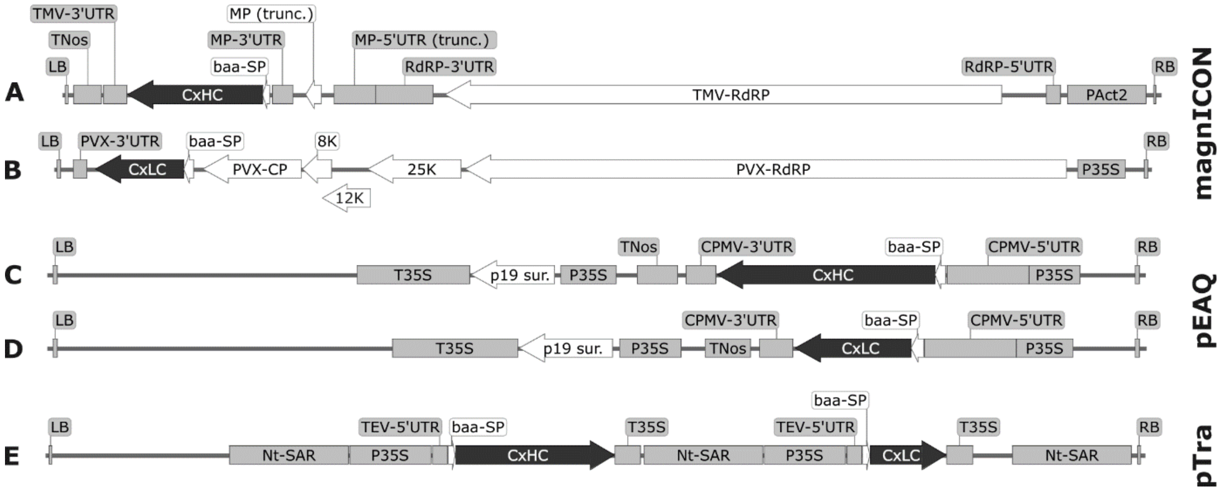

Supplementary Figure 1: Schematic presentation of relevant sequences between left and right border of transient expression vectors **(A,B) magnICON:** pICH26211-CxHC (10,882 bp), pICH31150-CxLC (7,931 bp), **(C,D) pEAQ:** pEAQ-HT-CxHC (6,755 bp), pEAQ-HT-CxLC (6,050 bp), **(E) pTra:** pMIDAS-CxHC-CxLC (9,281 bp). Figure was generated with SnapGene.

Supplementary Table 1: Detailed descriptions of abbreviated sequences of Suppl. Fig 1 in alphabetical order.

**8K, 12K, 25K**	viral proteins of Potato virus X
**baaSP**	signal sequence of barley alpha amylase
**CPMV-3’UTR**	3’-untranslated region of Cowpea mosaic virus
**CPMV-5’UTR**	5’-untranslated region of Cowpea mosaic virus
**CxHC, CxLC**	heavy and light chain of Cetuximab
**LB, RB**	left and right border
**MP (trunc.)**	truncated Tobacco mosaic virus movement protein
**MP-3’UTR**	3’-untranslated region of the Tobacco mosaic virus movement protein
**MP-5’UTR (trunc.)**	truncated 5’-untranslated region of the Tobacco mosaic virus movement protein
**Nt-SAR**	scaffold attachment region from *Nicotiana tabacum*
**p19 sur.**	p19 RNA silencing suppressor of Tomato bushy stunt virus
**P35S**	35S promoter
**pAct2**	Actin 2 promoter
**PVX-3’UTR**	3’-untranslated region of Potato virus X
**PVX-CP**	modified coat protein of Potato virus X
**PVX-PdRP**	modified RNA-dependent RNA-polymerase of Potato virus X
**RdRP-3’UTR**	3’-untranslated region of the Tobacco mosaic virus RNA-dependent RNA-polymerase
**RdRP-5’UTR**	5’-untranslated region of the Tobacco mosaic virus RNA-dependent RNA-polymerase
**T35S**	35S terminator
**TEV-5’UTR**	5’-untranslated region of Tobacco etch virus
**TMV-3’UTR**	3’-untranslated region of Tobacco mosaic virus
**TMV-RdRP**	modified RNA-dependent RNA-polymerase of Tobacco mosaic virus
**TNos**	nos terminator

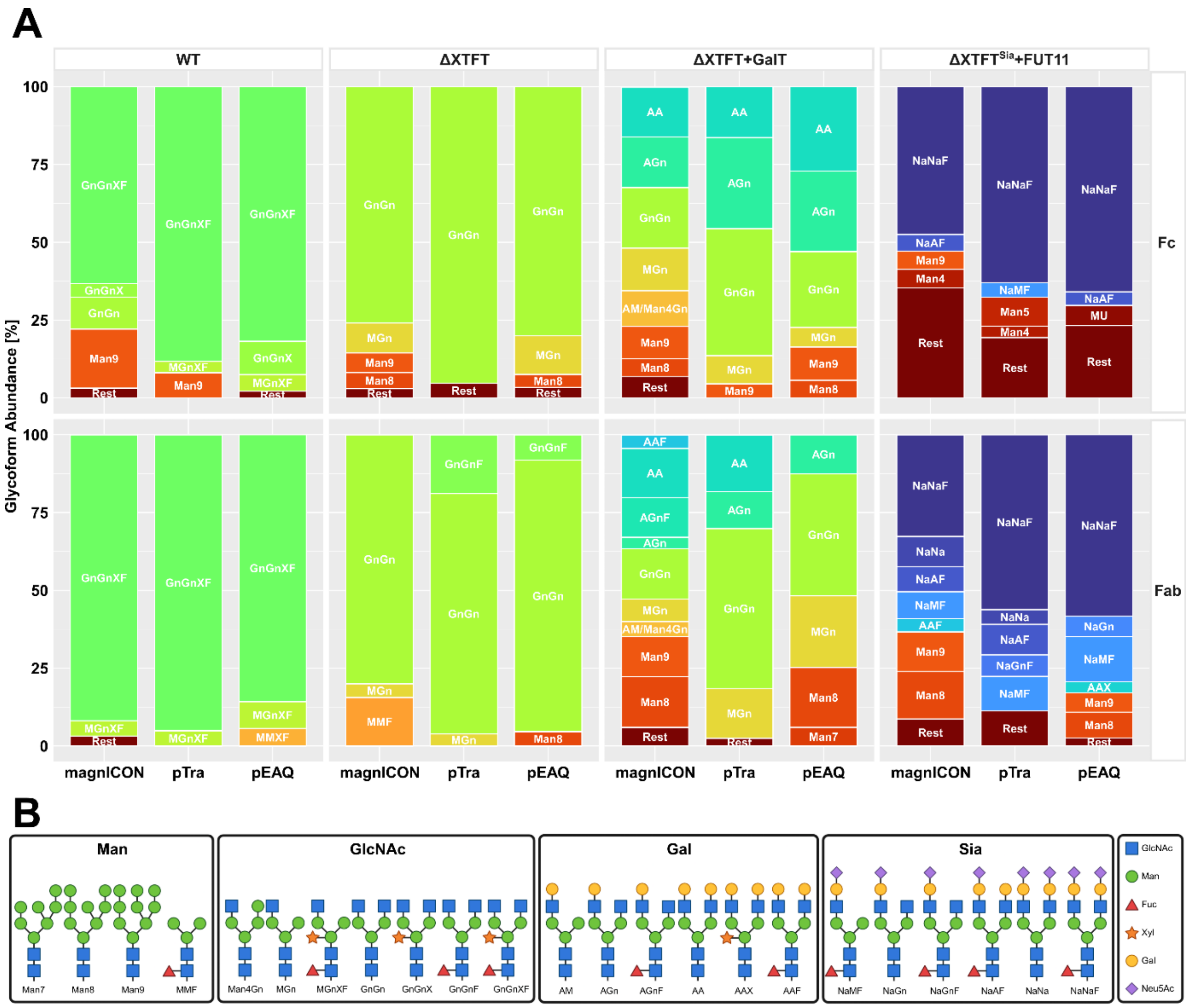

Supplementary Figure 2: **(A)** Detailed information on detected glycoforms using LC-ESI-MS analyses. Bars represent the relative abundance (in %) of glycoforms present at the Fc and the Fab domain of Cx expressed with magnICON, pTra or pEAQ modules; **(B)** Schematic presentation of most N-glycans found, grouped as in Fig 3. Low abundance glycoforms (<3.5 %) were subsumed as “Rest”. AM/Man4Gn percentage was incorporated into the GlcNAc fraction of Fig 3. Nomenclature according to Consortium for Functional Glycomics. (B) was generated with Biorender.

Supplementary Table 2: Utilized primers and restriction enzymes in cloning procedures. Restriction sites are underlined. pTra was constructed through two entry vectors (pBlue, pWhite (van Dollewerd, Kessans et al., 2018)). magnICON constructs were produced in past efforts (Castilho et al., 2015).

**Plasmid**	**Vector**	**Cloning technique**	**PCR primer**	**Restriction Enzymes**	**Citation**
**pMIDAS-*CxHC*-*CxLC***	MIDAS/ pTra	Restriction-Ligation; Golden gate	**HC**: TATACCATGGCGAACAAACACTTG and TATATCTAGATTACTTTCCAGGAGAAAGAGAA; **LC:** TATACCATGGCGAACAAACACTTG and TATATCTAGATTAGCATTCACCTCGATT	Restriction-Ligation: Nco1, Xba1; Golden gate: Bsa1, BsmB1	(van Dolleweerd et al., 2018)
**pEAQ-HT-*CxHC***	pEAQ	Restriction-Ligation	TATAACCGGTATGGCGAACAAACACTTG, TATACTCGAGTTACTTTCCAGGAGAAAGAG	Age1, Xho1	(Sainsbury et al., 2009)
**pEAQ-HT-*CxLC***		Restriction-Ligation	TATAACCGGTATGGCGAACAAACACTTG, TATACTCGAGTTAGCATTCACCTCGATTAAAG	Age1, Xho1	

Supplementary Table 3: Fc glycosite occupancy of magnICON-, pTra- and pEAQ-Cx expressed under different glycan engineering settings (represented in %).

**Vector**	**WT**	**ΔXTFT**	**ΔXTFT+Gal**	**ΔXTFT^{Sia}**	**Average**
**magnICON**	64	47	41	57	52
**pTra**	56	83	62	71	68
**pEAQ**	63	79	72	64	70

CxHC

```
ATGGCGAACAAACACTTGTCCCTCTCCCTCTTCCTCGTCCTCCTTGGCCTGTCGGCCAGCTTGGCCTCAGG
TCAGGTGCAGCTGAAGCAGTCAGGACCTGGCCTAGTGCAGCCCTCACAGAGCCTGTCCATCACCTGCACAG
TCTCTGGTTTCTCATTAACTAACTATGGTGTACACTGGGTTCGCCAGTCTCCAGGAAAGGGTCTGGAGTGG
CTGGGAGTGATATGGAGTGGTGGAAACACAGACTATAATACACCTTTCACATCCAGACTGAGCATCAACAA
GGACAATTCCAAGAGCCAAGTTTTCTTTAAAATGAACAGTCTGCAATCTAATGACACAGCCATATATTACT
GTGCCAGAGCCCTCACCTACTATGATTACGAGTTTGCTTACTGGGGCCAAGGGACTCTGGTCACTGTCTCT
GCAGCATCAACCAAAGGTCCTTCAGTCTTTCCACTTGCTCCTTCTTCAAAGTCGACTTCTGGCGGAACCGC
TGCCCTTGGATGTCTTGTTAAAGACTATTTCCAGAGCCAGTTACTGTCTCTTGGAACTCTGGAGCTCTTA
CTTCTGGCGTTCACACTTTTCCAGCTGTGCTTCAATCTTCTGGACTTTATTCTCTTTCCTCTGTTGTTACT
GTGCCATCTTCCTCTCTTGGAACTCAAACTTATATTTGTAATGTTAATCACAAGCCATCTAACACCAAAGT
GGATAAGAAAGCTGAGCCTAAGTCCTGTGATAAGACTCATACTTGTCCACCATGTCCAGCTCCAGAGCTTC
TTGGCGGACCATCTGTGTTCCTCTTTCCACCAAAGCCAAAAGACACTCTTATGATTTCTAGGACTCCAGAA
GTTACTTGTGTTGTTGTGGATGTTTCTCATGAAGATCCAGAAGTTAAGTTTAACTGGTACGTTGATGGAGT
GGAAGTTCATAATGCTAAGACTAAGCCAAGAGAAGAACAATATAACTCCACTTATAGAGTTGTGTCTGTTC
TTACTGTGCTTCACCAAGACTGGCTTAATGGAAAAGAGTACAAGTGTAAAGTGTCTAACAAAGCCCTTCCA
GCTCCTATCGAGAAGACTATTTCCAAAGCCAAGGGACAGCCTAGGGAACCACAAGTGTACACTCTTCCACC
ATCTAGGGATGAGCTTACTAAGAACCAAGTTTCTCTTACTTGTCTTGTGAAGGGATTTTATCCATCTGACA
TCGCCGTGGAATGGGAATCCAACGGACAACCAGAGAACAATTACAAGACTACTCCACCAGTTCTTGATTCT
GATGGATCCTTCTTTCTTTATTCCAAGCTTACTGTTGATAAGTCCAGATGGCAGCAAGGAAATGTGTTCTC
TTGTTCTGTTATGCACGAAGCTCTTCATAATCATTATACTCAAAAGTCCCTTTCTCTTTCTCCTGGAAAGT
AA
```

CxLC

```
ATGGCGAACAAACACTTGTCCCTCTCCCTCTTCCTCGTCCTCCTTGGCCTGTCGGCCAGCTTGGCCTCAGG
TGACATCTTGCTGACTCAGTCTCCAGTCATCCTGTCTGTGAGTCCAGGAGAAAGAGTCAGTTTCTCCTGCA
GGGCCAGTCAGAGTATTGGCACAAACATACACTGGTATCAGCAAAGAACAAATGGTTCTCCAAGGCTTCTC
ATAAAGTATGCTTCTGAGTCTATCTCTGGAATCCCTTCCAGGTTTAGTGGCAGTGGATCAGGGACAGATTT
TACTCTTAGCATCAACAGTGTGGAGTCTGAAGATATTGCAGATTATTACTGTCAACAAAATAATAACTGGC
CAACCACGTTCGGTGCTGGGACCAAGCTGGAGCTGAAACGTACTGTTGCAGCTCCTTCAGTGTTCATTTTT
CCACCATCTGATGAACAACTAAAGTCTGGAACTGCATCTGTTGTTTGTCTCCTAAACAATTTCTATCCCAG
AGAAGCTAAGGTTCAGTGGAAAGTTGACAATGCTCTTCAATCTGGAAACTCACAAGAGAGTGTTACTGAGC
AAGATAGCAAGGATAGCACATACTCTCTTTCTTCCACTCTTACTCTTTCCAAAGCTGATTACGAGAAACAC
AAGGTATACGCTTGCGAAGTGACTCATCAAGGACTTTCTAGTCCTGTTACAAAGTCCTTTAATCGAGGTGA
ATGCTAA
```

Supplementary Figure 3: DNA-sequences of expressed ORFs; grey: barley α-amylase signal sequence; green: variable domains of Cetuximab (Cx); orange: constant domains of IgG1; HC, LC: heavy chain, light chain.
